# Embryonic Hormetic Priming Modulates Later‐Life Thermal Tolerance

**DOI:** 10.1002/ece3.73139

**Published:** 2026-03-14

**Authors:** K. Lugue, C. J. Monaco, L. Benestan, E. Vigouroux, M. Sham Koua, J. Vidal‐Dupiol, G. Mitta, J. Le Luyer

**Affiliations:** ^1^ Ifremer IRD Institut Louis‐Malardé, Univ Polynésie française, UMR 241 SECOPOL Taravao Tahiti, Polynésie française France; ^2^ IHPE, Univ. Montpellier CNRS, Univ. Perpignan Via Domitia Montpellier France; ^3^ Ifremer Univ Brest, CNRS, IRD, UMR 6539, LEMAR Plouzané France

**Keywords:** developmental plasticity, marine, mollusks, RNA‐seq, thermal priming, transcriptomics

## Abstract

Understanding the mechanisms explaining thermal tolerance variation is crucial for predicting the impact of climate change on ectotherms, especially those living near their upper thermal limits. Among the various forms of plasticity, developmental plasticity holds promise as an adaptive trait for aquatic organisms to buffer the negative effects of ocean warming; however, its underlying molecular mechanisms remain poorly understood. Here, we examine the capacity of two bi‐parental progenies of the black‐lip pearl oyster, *Pinctada margaritifera* , to modify their later‐life thermal tolerance and performance through developmental thermal priming. Embryos (3–24 h post‐fertilization) were incubated until hatching at either control (28°C) or warm (32°C; *ecological extremes*) temperatures, and raised 4 months under common conditions at 28°C. Our results reveal family‐specific effects of early‐life thermal priming, significantly enhancing spat thermal tolerance in one family, while reducing it in the other. Main molecular pathways of heat stress response (at the sublethal temperature of 34°C) were conserved across families and independent of the early‐life priming treatment. Nevertheless, a network‐preservation approach allowed further characterizing the subtle, nested environmental “memory” mediated through network reorganization, particularly in gene regulatory pathways involved in the Unfolding Protein Response (UPR). While this 2 × 2 factorial design may capture an oversimplification of the complex relationship between early environments and later phenotypes, our study emphasizes the need to refine and develop new well‐tuned experimental protocols to hold out the promise of hormetic priming for ecological conservation and aquaculture improvement.

## Graphical abstract

This study investigates how early‐life thermal environments affect later thermal tolerance in the black‐lip pearl oyster, *Pinctada margaritifera* . Developmental thermal priming had contrasting, family‐specific effects on thermal tolerance, with conserved stress‐response pathways but evidence of priming‐induced regulatory network reorganization. These findings highlight the potential and complexity of developmental plasticity for adaptation to ocean warming.

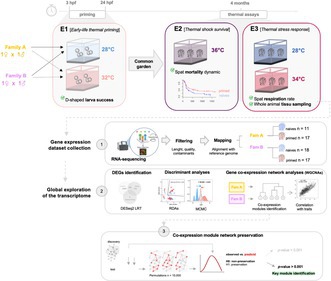

## 1 Introduction

The thermal tolerance of organisms, i.e., thermal performance optima and limits, is genetically determined, but also subject to plasticity controlled by multiple non‐genetic processes (Thomson et al. 2018). In an era of unprecedented climate change, identifying the potential factors driving thermal tolerance variations is crucial, especially for tropical and sessile ectotherms already leaving close to their upper thermal limits (Deutsch et al. 2008; Dillon et al. 2010; Vinagre et al. 2019).

Phenotypic plasticity, the ability of a genotype to produce different phenotypes in response to distinct environmental conditions, is a universal property of living organisms and has been identified as a process that can contribute to species adaptive capacity to cope with climate change (Chevin et al. 2010; Hoffmann and Sgrò 2011). In thermal biology, beneficial plasticity, often referred to as *acclimatization* or *acclimation*, is characterized by changes in the shape/slope of the relationship between traits (e.g., performance, upper thermal limits) and environmental drivers (Huey et al. 1999; Seebacher et al. 2015). In aquatic organisms, physiological adjustments can enhance thermal tolerance by several degrees (e.g., see Earhart et al. 2023). These physiological adjustments are evident at both the whole‐organism level (e.g., metabolic rate, size of organs and tissues; Sinclair et al. 2016) and the cellular level (e.g., cell membrane, changes to organelles, heat shock proteins; Cleves et al. 2020; Zhang and Dong 2021). However, the maintenance and production costs (DeWitt et al. 1998) represent a major limitation for the long‐term persistence of such responses, which can vary from hours (Bowler 2005; Sørensen et al. 2019) to months (Drury et al. 2022; Moyen et al. 2020). Despite recent progress in our understanding of the underlying molecular mechanisms driving variation in thermal tolerance plasticity, much of the knowledge comes from studies on juvenile and adult organisms, with relatively little attention given to the processes occurring during early‐development life stages although considered as the most sensitive to environmental stresses (Dahlke et al. 2020).

The developmental period of an organism is sensitive to changes in the surrounding environment (Byrne et al. 2020; Collin et al. 2021; Dahlke et al. 2020; Pandori and Sorte 2019; Truebano et al. 2018). Numerous studies have examined the effects of developmental stressors (e.g., deleterious effects of increasing temperature and acidification on fertilization success of marine invertebrate species; Byrne 2011). Stress exposure at early‐life stages can, however, induce long‐lasting effects on later‐life phenotypes including juveniles and adults (Bateson et al. 2014). Studies on ectothermic organisms have shown that early‐stress increases later life mean thermal tolerance (Schnurr et al. 2014) as well as acclimatization capacity (Beaman et al. 2016; e.g., see in copepods: Healy et al. 2019; zebra fish: Scott and Johnston 2012). These results fall in the hormetic priming hypothesis, which posits that low or sublethal doses of a stressor provide physiological benefits (Costantini et al. 2010; Gems and Partridge 2008). This adaptive response is driven by developmental plasticity, which can result from both short‐ (*developmental priming*) or long‐term exposure (*developmental acclimation*). Still, contrasting responses have been described either because of the use of different taxa or because of differences between thermal stress characteristics across studies—drawing controversial conclusions about the implication for survival in a climate change context. For example, Kellermann and Sgrò (2018) found that elevated developmental temperatures negatively affected the later‐life acclimation response of tropical fruit fly species, whereas subtropical species exhibited a positive effect. Similarly, Glass et al. (2023) observed that early‐life thermal priming had negative effects on sea anemones' heat tolerance under high‐intensity priming treatment, but produced positive effects under milder priming treatments. These discrepancies highlight the need to better understand persistent effects of developmental thermal plasticity by deciphering the underlying molecular mechanisms.

Organisms respond to their environment (stress) through changes in gene expression, over a period of minutes to hours (Wagner 2018), reflecting acclimatory responses to maintain cellular and molecular homeostasis. Influenced by both genotype and environment, gene expression data enable comparisons across individuals, offering insights into the molecular mechanisms underlying individual phenotypes (Feiner et al. 2018). Transcriptomic profiles are commonly explored through differential gene expression analyses; however, complex traits are expected to be polygenic (Boyle et al. 2017) and is often difficult to discriminate between true‐ and false‐positive candidate genes, especially when individual genetic variation is subtle and many genes are potentially involved. Such dimensionality issue can be addressed using analysis focusing at the upper organizational (i.e., the biological process) level accounting for the sum of effect of several genes (de Lorgeril et al. 2020) and correlative approaches (e.g., co‐expression networks), although differences are commonly flattened when building consensus networks (i.e., over multiple states, conditions, etc.; Chevin et al. 2022). While numerous studies have described DNA methylation in early‐life primed ectotherms (i.e., warmer embryonic environment; Anastasiadi et al. 2017; Fellous et al. 2015; Loughland et al. 2021), very few have revealed differences in gene expression, mostly relying on pairwise comparisons (Metzger and Schulte 2018; Scott and Johnston 2012) or consensus network (Gurr et al. 2022) analyses. Recent advance in computational power and analytical frameworks that subdivide large datasets into biologically relevant clusters, have allowed successfully detecting gene network architectures related to disease (Iacono et al. 2019; Tommasini and Fogel 2023). This framework was used in Ripley et al. (2023) to assess species‐ and early‐life environment‐specific transcriptional network entropy, opening up a promising avenue to improve our understanding of the underlying biology in developmental plasticity.

Leveraging early conditioning protocols to optimize husbandry under ocean warming, while preserving population genetic diversity, constitutes a key challenge for the future of aquaculture. Here, we thus investigated whether thermal priming during early development stages affects later‐life thermal performance, and how such a response might be underpinned by molecular memory. We used a common‐garden experimental approach to examine molecular responses of two bi‐parental progenies exposed to a thermal priming treatment during the early sensitive life window of embryogenesis (3–24 h post‐fertilization). After scoring for later‐life phenotypes, we assessed the effect of thermal priming on the gene‐expression profiles/levels by conducting differential expression and co‐expression network analyses in association with subsequent enrichment analysis for each full‐sib family separately. Then, we employed permutational preservation analyses to explore gene network architectures (gene–gene connections) conservation or divergence across families. On the basis of the hormetic priming hypothesis, we hypothesized that primed individuals would exhibit higher tolerance to heat stress, compared to naïve individuals. We also predicted that enhanced thermal tolerance would be associated with transcriptome modulation and network restructuration.

## 2 Materials and Methods

### 2.1 Experimental Animals

In this study, we used the marine tropical bivalve *Pinctada margaritifera* as a model species. In the literature, *P. margaritifera* is most often described as a subtidal bivalve inhabiting lagoon environment, typically at depths of 20–30 m, where environmental conditions are assumed to be relatively stable. However, archival records indicate that pearl oyster fishing in the early 19th century was largely limited to the collection of individuals in waist‐deep waters (Intes 1982). These historical observations, together with frequent contemporary reports of *Pinctada* species occurring in shallow habitats (e.g., Derbali et al. 2019; Reisser et al. 2019), suggest that the ecological distribution of *P. margaritifera* may be broader than traditionally acknowledged and not strictly confined to subtidal environments.

Marquesan breeders collected from tide pools (Ua Pou; French Polynesia; 9°25′18.7″ S, 140°03′07.5″ W) were transferred to Ifremer's marine concession in Vairao (Tahiti, French Polynesia; 17°48′35.5″ S, 149°17′54.5″ W) in October 2015. During this acclimatization period in Vairao, no temperature overpassing species thermal breath was recorded. In January 2022, breeders (two males and two females) were transferred to controlled experimental facilities, in order to initiate a synchronized gametogenesis. Oysters bi‐parental reproductions were conducted on August 17, 2022 (hereafter referred to as family A) and September 21, 2022 (hereafter referred to as family B) at the Ifremer facility for logistic constraints. Breeders husbandry, spawning induction, and larval rearing followed identical standard procedures (Destanque et al. 2024). Briefly, from D‐shape stage to pediveliger, larvae were fed ad libitum with a mixture (1:1) of *Isochrysis galbana* and *Chaetoceros minus* and maintained in throughflow 50 L tanks system in 1 μm and UV‐treated water at ambient temperature (mean 28°C) with upwelling oxygen supplementation. Larvae were sieved at day 13 and day 19 on 60 and 80 μm mesh, respectively, to remove any deposited organic material. At the pediveliger stage, larvae were transferred to a micronursery through‐flow raceway system using 5 μm‐ and UV‐treated seawater at ambient temperature. They were maintained for up to 5 months under common garden conditions (i.e., same raceways across families and developmental treatments) and fed a 1:1 mixture of *Isochrysis galbana* and *Chaetoceros minus*.

### 2.2 Experimental Design

**FIGURE 1:**
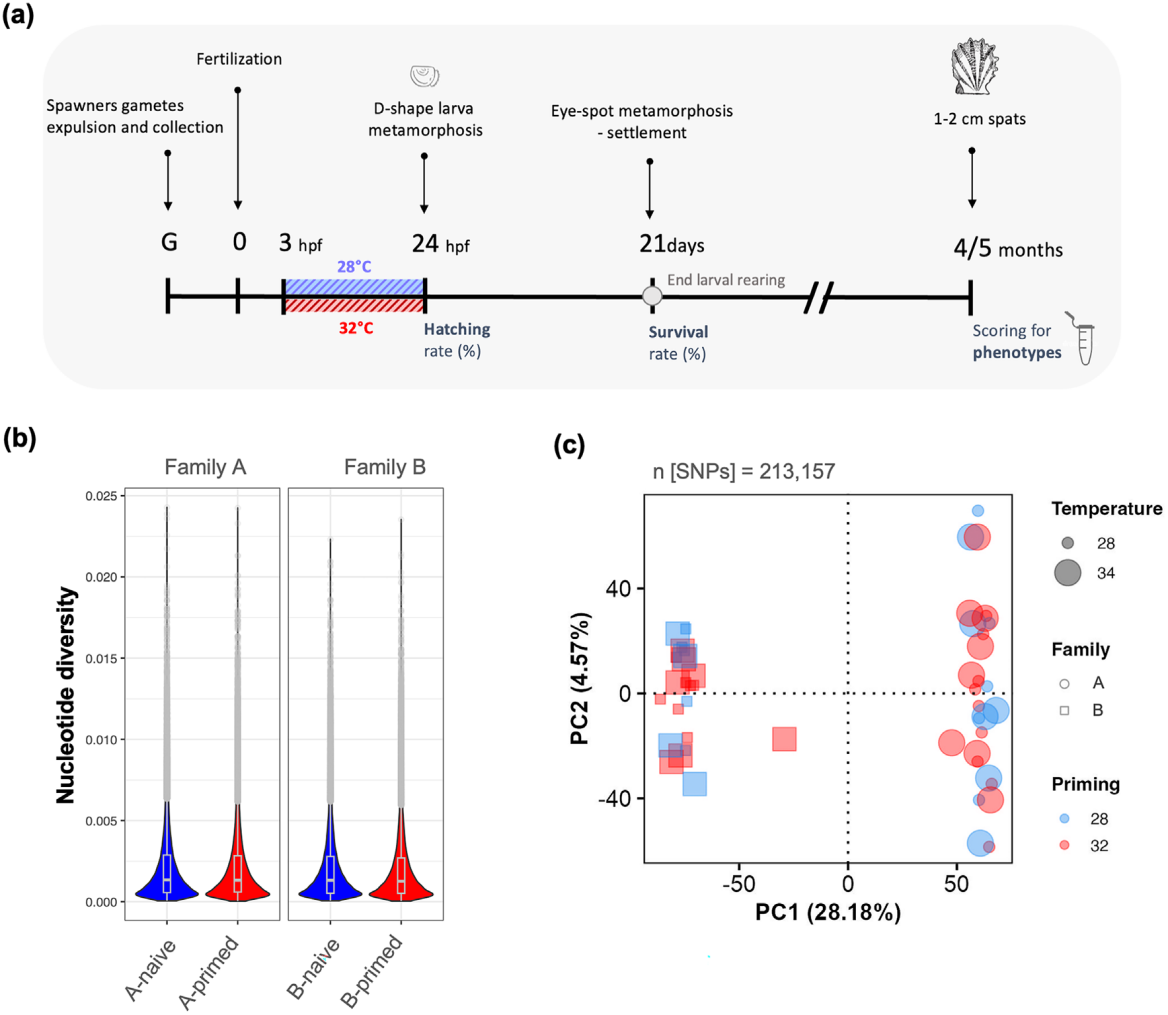
Early‐life thermal priming on *Pinctada margaritifera* embryos. (a) Schematic representation of the experimental design. Early thermal primed individuals (32°C) are represented in red, and naive individuals (28°C) are represented in blue. (b) Estimated of nucleotide diversity (Π) based on windows size of 100 kb. (c) PCA of the genomic variation (*n* = 213,157 SNPs).

Using controlled pedigree and bi‐parental families with known life‐history traits, we exposed *P. margaritifera* larvae to either a control condition (28°C) or early‐life thermal stress (32°C) for the first 3 to 24 h post‐fertilization (i.e., early life thermal priming; see Section 2.2.1). The stressful thermal treatment was selected based on previous experimental work and its ecological relevance, as it decreases D‐shaped relative survival, and reflects thermal extremes occasionally experienced in lagoon environments (Lugue et al. 2025). After 4 months of common‐garden conditions, we quantified spat thermal tolerance using a lethal thermal stress assay (see Section 2.2.2) and thermal plasticity, along with associated molecular responses, using a sublethal thermal stress assay (see Section 2.2.3). These measurements allowed us to assess the effect of early‐life thermal priming on later‐life thermal phenotypes (Figure 1a and Figure S1).

#### 2.2.1 Early‐Life Thermal Priming

Upon spawning, the gametes from each breeder were immediately isolated (placed separately in a plastic container, and their quality judged based on eggs appearance and sperm mobility), before pooling. At 3 h post‐fertilization (hpf), zygotes were divided equally and incubated in 70 L plastic containers. On the basis of natural seasonal maxima (Bruyère et al. 2023), two temperature regimes were applied in duplicate: control (28°C) vs. stress developmental temperature (32°C), until the D‐shape stage was reached (~24 hpf; i.e., “incubation”). One the larval stages completed, progenies for each family (naïve vs. primed) were further maintained in a common‐garden, under hatchery‐controlled conditions (see details in Data S1), until the experiments began (i.e., 4 months later).

#### 2.2.2 Scoring Spats Thermal Tolerance

To assess differences in thermal tolerance between cohorts, juveniles aged of 4 and 5 months (*n* = 240), were sampled from the common garden for the family A and B (same fresh weight condition across families with a mean Fresh Weight [FW] = 0.19 ± 0.06 g; mean Length [L] = 13.42 ± 1.52 mm; Figure S3) and assigned to independent experimental tanks (*n* = 30 individuals in duplicate tanks, see details in Data S1). At this time, spats share similar ontogenic stages, i.e., post‐metamorphic and pre‐reproductive stage, with only male individuals (hermaphrodite protandrous species with sex conversion starting around 2 years old; Chávez‐Villalba et al. 2013). Spats were allowed to acclimate for 4 days, at the control mean temperature of 27.29°C ± 0.79°C, during which no mortality was recorded. Then, animals were exposed to a progressive increase in temperature (+1.3°C h^−1^), reaching 36°C in 6 h. The 36°C condition corresponds to a lethal thermal condition, leading to LT50 mortality within few days for the species (Lugue et al. 2025) and elevated heating rate preventing acclimation process to occur. Oysters were maintained at this static target temperature until mortality in the tanks reached 100%. Oyster status (alive/dead) was checked every 30 min during the initial 24 h, and every 2–3 h thereafter. Assessment was done visually and by mechanical stimulation (gently poking the mantle) following previous protocol (Lugue et al. 2025). When animals did not respond to stimuli (valves were opened and did not respond to contact), time was recorded and the individual was removed from the tank. The lethal temperature for 50% survival (LT50) was recorded for each tank.

The survival‐time curves of oysters exposed to heat shock (36°C) were compared using the mixed Cox regression model (coxme v2.2–20 R package; Therneau 2024); considering fixed (family, priming, and their interaction) and random (Tank) effects. The proportionality of hazards (PH) was checked with martingale residuals (Lin et al. 1993). Significant differences between replicates were investigated using the *survdiff()* function of the survival v3.6–4 R package (Therneau and Grambsch 2000). Depending on whether the survival curves crossed or not, either the Wilcoxon test (rho = 1) or the log‐rank test (rho = 0) was applied, respectively. Survival rates were represented as Kaplan–Meier curves using the survminer v0.4.9 R package (Kassambara et al. 2021).

#### 2.2.3 Scoring Spats for Thermal Plasticity

In a second experiment, we assessed whether cohorts had divergent thermal responses to a sublethal stress. Spats, from both families (A and B, *n* = 200 per family) were collected from the control common garden condition (2023.01.30; mean FW = 0.20 ± 0.05 g; mean L = 129.6 ± 16.0 mm; Figure S4), and subjected to two thermal conditions: control (28°C) vs. heat stress (34°C). The 34°C condition corresponds to the critical thermal limit, delineating the permissive (i.e., life completion) from the stressful (i.e., mortality) range for this species (Le Luyer et al. 2022; Lugue et al. 2025). This temperature induces a thermal response without causing mortality during the experimental period or over subsequent weeks, thereby ensuring that the sampled individuals did not show sign of physiological collapse (Le Luyer et al. 2022; Lugue et al. 2025). Each combination family × priming × temperature was set in independent experimental tanks (*n* = 50 individuals per tank), and duplicated. Animals were allowed to acclimate for 4 days, at the control mean temperature of 28.04°C ± 0.57°C, before the different treatments were applied. On day 0 (before the start of the experiment), 5 animals from each experimental combination were sampled (whole individual), and preserved in RNA‐Later for 24 h at 4°C, then stored at −80°C until subsequent transcriptomics analyses. The heat‐stress condition included a progressive increase in temperature (+1°C h^−1^ over 6 h), to reach a maximum plateau at 34°C. After 24 h of exposure, five oysters were individually sampled from each tank, preserved in RNA‐Later, and stored at −80°C. On days 3 and 4, respiration rates were measured on individuals from the control (28°C) and the heat stress (34°C) conditions (*n* = 5 replicates per combination), using a closed‐system respirometry approach (see details in Data S1). Briefly, individuals of similar weight were starved 24 h prior starting the measurements that were run for a maximum of 1 h and half to prevent O_2_ levels to drop below normoxic conditions and animals were observed for this total duration to ensure no valves closing. Respiration rates (RR; mg O_2_ h^−1^) were corrected to a standard 1‐g animal, using the formula:

(S1) $$Ys={\left(Ws/We\right)}^{b}\times Ye$$

where *Y*s is the corrected metabolic rate, *W*s is the standard dry weight (1 g), *W*e is the measured dry weight, *Y*e is the measured physiological activity, and *b* is the allometric coefficient. On the basis of Savina and Pouvreau (2004), we used the *b* allometric coefficient of 0.75. Note that to avoid biasing the transcriptomic analysis with the starving process, respirometry was conducted on a separate set of individuals sampled from the same tanks as the transcriptomics.

### 2.3 RNA Extraction

Total RNA was extracted from the whole individual. Cellular lysis was induced with 1 mL of TRIzol, grinded under ball mill and according to manufacturer protocol. Briefly, after centrifugation at 12,000× *g* for 10 min at 4°C, the lysate was then transferred into a 1.5‐mL tube. The phase separation was achieved by adding 200 μL of chloroform followed by centrifugation at 12,000× *g* for 12 min at 4°C. RNA was precipitated from the aqueous phase by adding 250 μL of isopropyl alcohol and high salt solution (for 1 mL of Trizol). Total RNA was finally washed with 900 μL of 75% ethanol, and resuspended in 90 μL RNase‐free water, before further quantification (NanoDrop ND‐2000 spectrophotometer; Thermo‐Fisher, USA), and quality‐control (Bioanalyzer; Agilent, USA) measurements. High‐quality RNA was sent to Genome Quebec sequencing platform (Montréal, QC, Canada) for mRNA‐stranded library preparation and sequencing (Illumina NovaSeq6000 S4 150 bp paired‐end).

### 2.4 RNA‐Seq and SNP Data Processing

Detailed pipeline and codes of RNA‐seq and SNP analyses are available online (https://gitlab.com/jleluyer/pinctadapt_priming). Raw reads were first filtered to ensure a minimum size (60 bp), a minimum quality (trailing = 20; leading = 20), and for potential contaminants/remaining adaptors (UniVec) using Trimmomatic v.0.36 (Bolger et al. 2014). Filtered reads were then mapped against the reference genome (Le Luyer et al. 2019) using STAR v 2.7.10b (Dobin et al. 2013), allowing for a maximum number of mismatches per pair of 10, a maximum number of multiple alignments of 1, a minimum and maximum intron length of 20 and 500,000, respectively. High quality and uniquely mapped reads were selected using Samtools v1.17 (Li et al. 2009; SAM flag 4 and 256, skip alignments higher than 5), and the final count matrix done using HTSEQ v0.6.1 (Anders et al. 2015).

To search for variants (single nucleotide polymorphisms; SNPs), mapped reads were processed using GATK v4.4.0.0 following recommendations for RNA‐seq data with prior removal of duplicated reads (Brouard et al. 2019). Raw dataset was subset for only bi‐allelic markers, missingness (> 10% missing data), minor allele frequency (MAF < 1%), minimum depth coverage of 10 and minimum quality of 30 with VCFtools v0.1.16 (Danecek et al. 2011). Imputation of the filtered markers (~10% missingness) was processed with Beagle v4.0 (Browning and Browning 2016).

### 2.5 Genomic Statistics and Variation

Nucleotide diversity was first explored by segments of 100 kb using *–windows‐pi* function implemented in VCFtools v0.1.16 (Danecek et al. 2011), for each family separately. We then compute a Euclidean distance matrix using the overall genomic variation, prior to run a principal coordinate analysis (PCoA; stats v4.4.1 and ape v5.8 R package). A distance‐based RDA (db‐RDA) with retained PCo factors (*n* = 3) and priming, families and temperature as explanatory variables using a stepwise model selection approach (partial db‐RDA). Outlier detection was also explored using PCAadapt (Luu et al. 2017). Finally, to detect possible genetic signatures of Treatment we tested 55 machine‐learning algorithms implemented in BioDiscML (Leclercq et al. 2019). Models were evaluated based on different evaluation procedures including 10 CV, leave‐one‐out cross‐validation (LOOCV), holdout, repeated holdout, bootstrapping, and 0.632+ bootstrap estimator. The overall best model was selected based on the receiving operating curve (ROC) and the area under the ROC curve (AUC) criterion.

### 2.6 Differential Gene Expression Analysis

Genes with low‐abundance transcripts (i.e., < 1 Count Per Million, CPM; in at least four individuals, representing approximately 50% of the population in each treatment) were removed, resulting in a 20,205 gene set. The final number of individuals retained for gene expression analyses were: 11 for FamA‐naive, 17 for FamA‐primed, 18 for FamB‐naive, and 17 for FamB‐primed. Gene counts were normalized using a (blind) variance stabilizing transformation (vst) from which a db‐RDA was performed to identify the dominant factors driving transcriptional variation. The selection of the optimal number of PCoA axes was done using the relative eigenvalues approach (Legendre and Legendre 2012). The DESeq2 v1.40.2 R package (Love et al. 2014) was used to statistically assess differential gene expression. We considered three models with likelihood‐ratio tests (LRTs) to test the effects of priming (naïve vs. primed), temperature (control vs. heat stress), and their interaction—for each family separately (A vs. B). We further explored differences in gene expression through contrasts, grouping the variables of early‐life environment and later‐life temperature exposure (i.e., naïve‐control vs. naïve‐stress, and primed‐control vs. primed‐stress; WALD test) within each family. For all likelihood‐ratio tests and contrasts, we applied a false discovery rate (FDR) adjusted *p*‐value of 0.01.

### 2.7 Weighted Gene Co‐Expression Network Analyses

We used the weighted gene co‐expression network approach, implemented in WGCNA v.1.72–5 R package (Langfelder and Horvath 2008) to build discrete signed networks for each family. First, we removed low‐variance genes (variance < 0.05) from the normalized expression matrix. Next, we built a weighted adjacency matrix with soft threshold power of 6 to reach a correlation coefficient R of 0.79 and 0.83, for family A and B, respectively. Outlier individuals were identified and removed based on a z‐score distribution connectivity threshold (i.e., < 2.5). A total of 28 and 23 individuals remained after filtration 18,932 and 18,958 genes for family A and B, respectively. We then performed hierarchical clustering of genes to identify groups with co‐varying expression patterns across samples. The minimum cluster size and merging similar (gene‐sharing) module threshold were set at 50 and 0.25, respectively. The expression of each module was summarized as a *Module Eigengene* (i.e., ME), calculated as the first principal component of all the genes within the module. Finally, these modules were correlated with factors and phenotypic traits including developmental temperature conditions, hereafter called priming, experimental temperature condition, hereafter called treatment, and individual weight, to determine module‐trait Spearman correlations.

### 2.8 Co‐Expression Gene Network Module Preservation

To explore if divergence in the effect of priming across families was not only linked to gene‐level modulation but also to a reorganization of the co‐expression matrices, we assessed module preservation through different metrics (i.e., network coherence, average and concordance of nodes contribution, density, and concordance of correlation structure, average edge weight and concordance of weighted degree) with permutation (*N* = 10,000) implemented in NetRep v1.2.7 R package (Ritchie et al. 2016). In this study, this step was done twice: for family B (i.e., discovery dataset) preservation in family A (i.e., test dataset; FamB‐A); and for family A (i.e., discovery dataset) preservation in family B (i.e., test dataset; FamA‐B). Strong evidence of module preservation in the other family was indicated when the permutation test *p*‐value was < 0.0001 (for a given module statistic). A module was considered unpreserved when the permutation test *p*‐value was < 0.0001 (significance threshold based on Ritchie et al. 2016).

### 2.9 Identification of Candidate Genes

In order to finely elucidate the underlying mechanisms of divergent thermal responses and identify putative candidate genes; a subset of genes was selected from each of the key modules (i.e., not preserved modules). To do so, we combined clustering and differential analyses to keep (i) genes the most correlated with the priming treatment based on gene significance (GS), and (ii) genes with the most afferent connections, respectively. Indeed, genes that are highly correlated with a large number of genes (“hub” genes) may be of particular importance in preserving the co‐expression networks. The top 1% of genes most strongly correlated with the identified candidate genes were then modeled, based on the adjacency matrix of the key module. Correlation was prior binarized, such that strong positive correlations was retained (only top 10% quantile of r values were retained, and weak correlations were discarded). Undirected networks were modeled using igraph v1.2.2 R package (nodes: genes, and edges: correlations of 1) and the list of nodes and edges imported in Gephi (v0.10.1, 2023) for sub‐network visualization.

### 2.10 Functional Enrichment

KOG (cluster of eukaryotic Orthologous Genes) classes of significant genes from the contrast analysis (DESeq‐derived log‐fold‐changes from WALD tests, see Section 2.8) were extracted from eggNOG‐mapper v2 (Cantalapiedra et al. 2021). Enrichment of each KOG class was done using a two‐sided Mann–Whitney *U* test, based on their log2 fold‐changes (KOGMWU v1.2 R package; Dixon et al. 2015). To test whether KOG delta ranks were correlated between families and early‐life environments, pairwise Pearson correlations were calculated and visualized using the corrplot v0.92 R package. Gene Ontology (GO) annotation of the reference genome was done by blasting (BLAST v2.12) nucleotide sequences to the SwissProt‐UniProt database (https://www.uniprot.org), allowing a maximum of five alignments, and retaining hits with a minimum of 10^−5^ e‐value. GO enrichments were performed on genes from signed WGCNA modules (kME value; remaining genes annotated as zero) using Fisher's exact test (absValue = 0.001; GO_MWU; https://github.com/z0on/GO_MWU). GO terms with corrected *p*‐value < 0.01 were considered significantly enriched.

## 3 Results

### 3.1 Early‐Life Thermal Challenge

Larval rearing performance differed between families and incubation conditions (see Figure S2 and Table S1 in Data S1). Rearing success was quantified using hatching rate (number of D‐shaped larvae relative to the number of initial embryos; 24 hpf) and survival rate (number of pediveliger larvae relative to the number of D‐shaped larvae; 21 dpf). In family A, hatching rates at 24 hpf ranged from 44% to 65% in naive and primed cohorts, respectively, whereas lower hatching success was observed in family B (33% in naive and 8% in primed cohorts). In contrast, post‐hatching survival was higher in family B, reaching 27% in naive progeny and 11% in primed progeny (see Figure S2 and Table S1 in Data S1). In family A, post‐hatching survival was reduced, declining from 17% to 2%–5% in naïve and primed cohorts, respectively. Although the contrasted patterns of early mortality (family B) vs. late mortality (family A) observed in the primed cohorts warrant attention, these values remain within the range typically reported for the genus across a variety of rearing protocols (Doroudi et al. 1999; FAO 1999; Saucedo et al. 2005; Southgate and Ito 1998; Utami et al. 2024).

### 3.2 Spat Survival and Respiration Rates

**FIGURE 2:**
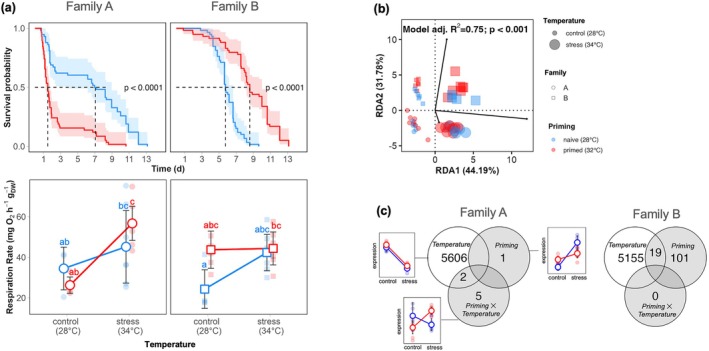
Long‐lasting effect of early‐life thermal priming on *Pinctada margaritifera* juveniles. (a) Upper panels represent effect of lethal thermal shock (36°C) on the survival of 4 months‐old spats. Bottom panels represent the effect of sublethal thermal stress (34°C) on the respiration rates of 4 months‐old spats (mean ± 95 CI). Early thermal primed individuals (32°C) are represented in red, and naive individuals (28°C) are represented in blue; for the family A (left panels) and B (right panels). Significance codes: ‘***’ 0.001 ‘**’ 0.01 ‘*’ 0.05. (b) Redundant discriminant analysis (RDA) of transcriptomic profiles (20,205; normalized count matrix) across families, early‐life backgrounds and later‐life temperature exposure. (c) Venn diagram of Differentially Expressed Genes (DEGs) obtained by likelihood ratio tests (LRTs); after correcting for FDR (< 0.01), for the family A and B, respectively.

Naïve groups showed no significant differences in thermal tolerance at 36°C (family A vs. B; post hoc emmeans: *p*‐value = 0.06; Figure 2a). However, the effect of priming depended on family (ANOVA: chi‐squared = 33.39, df = 1, *p*‐value < 0.001). Indeed, Lt50 for spat survival in family A (Lt50 = 1 day) was significantly reduced by priming (post hoc emmeans: *p*‐value < 0.001), while priming significantly enhanced thermal tolerance in family B (Lt50 = 9 days; see Figure 2a).

The effect of priming on respiration rate also varied with family and temperature (ANOVA: df = 1, *F* = 9.42, *p*‐value < 0.01). While temperature had no effect on respiration rates in family B, the primed cohort of family A exhibited significantly lower respiration rates under control conditions (28°C) compared to heat stress (34°C) (post hoc emmeans: df = 44, *p*‐adjusted < 0.001; Figure 2a).

### 3.3 Genomic Variation

A total of 213,157 bi‐allelic SNPs were retained after filtering, with an average of 10% missing data prior to imputation. Family alone explained a significant 37% of the genomic variance, while controlling for the effects of priming and temperature (ANOVA; partial db‐RDA; *F* = 151.10; *p* < 0.001; Figure 1b). In contrast, priming and temperature had no significant effect on SNP frequencies (ANOVA; partial db‐RDA; *p* = 0.70 and *p* = 0.47, respectively). Besides, no differences in nucleotide diversity were observed across groups, suggesting no evident genetic bottleneck or directional selection. Furthermore, BioDiscML detected only a small number of significant outliers associated with priming—eight in family A (Normalized Poly Kernel model; bootstrapping accuracy = 0.945, 10‐fold cross‐validation = 1) and five in family B (Logistic model; bootstrapping accuracy = 0.957, 10‐fold cross‐validation = 0.937). Importantly, priming did not contribute to the explained variance in the RDA models. Together, these results indicate that selection was either absent or very weak during the early‐life challenge and not associated with thermal preferences, despite the high mortality observed, and that the differences in thermal tolerance likely reflect nongenetic (e.g., plastic or stochastic) variation.

### 3.4 Transcriptomics Profiles

The distance‐based Redundancy Analysis (db‐RDA) model for the whole transcriptome expression profiles, which included family, priming, and temperature, explained 74.97% of the total variance (db‐RDA; ANOVA; *F* = 54.73; *p*‐value < 0.001; Figure 2c). Temperature alone accounted for 44.19% of the variance explained by the model, while controlling for the effects of family and priming (ANOVA; partial db‐RDA; *F* = 64.77; *p*‐value < 0.001). Family alone accounted for 31.78% of the overall transcriptomic variance (ANOVA; partial db‐RDA; *F* = 32.47; *p*‐value < 0.001). The priming effect was significant in the global RDA model; yet, when controlling for family and temperature, the effect was no longer significant (ANOVA; partial db‐RDA; *F* = 0.98; *p*‐value = 0.35), suggesting that priming is context‐dependent.

### 3.5 Differentially Expressed Genes (DEGs)

We found 5608 and 5174 differentially expressed genes between the control and heat‐stress temperatures (28% and 26% of the transcriptome) for the family A and B, respectively (FDR < 0.01). Full models including priming and temperature (without interaction term) were significant for one transcript in family A, and for 120 transcripts in family B. Only seven genes, in family A, were significantly explained with the model including the interaction term. DEGs overlap for each family, is shown Figure 2c. DEGs of interest—i.e., significantly responsive to the priming and priming × temperature—were found to be implicated in processes such as transcription (e.g., kinase protein, transcription factor, etc.), ion transport (e.g., calcium, sodium, etc.), or protein turnover (e.g., thiol protease). All annotated, differentially‐expressed genes can be found in Table S1. Enrichment of each KOG class with up‐ or down‐regulated genes from the contrast analysis is presented in Figure S5.

### 3.6 Family‐Wise Weighted Gene co‐Expression Network Analysis

**FIGURE 3:**
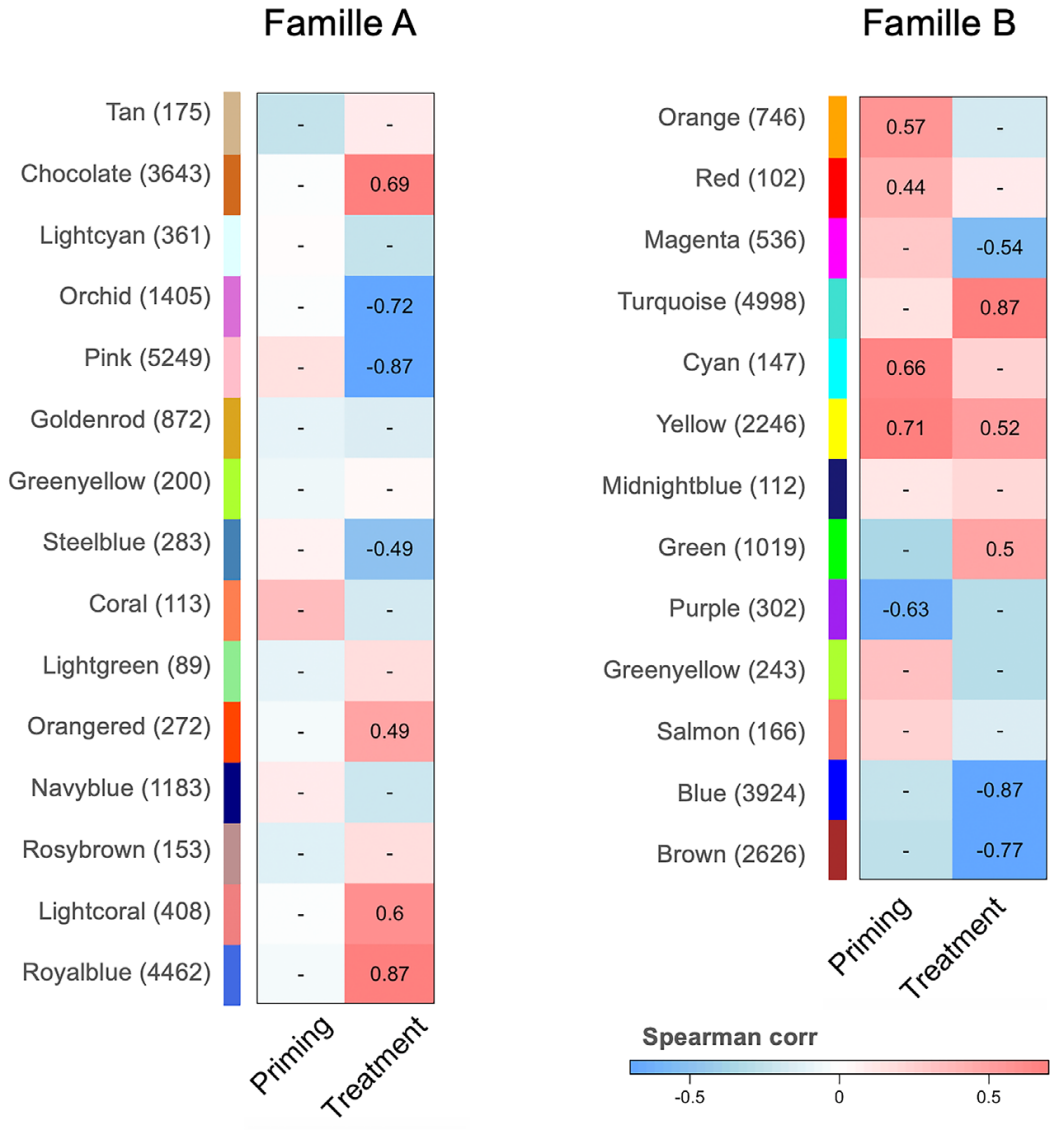
Transcriptome modulation. Heatmap of identified modules (*y*‐axis) significantly correlated with traits (*x*‐axis; Spearman method; *p*‐value < 0.05) from WGCNA, for the family A and B. The number of genes found within each module is indicated in parentheses, and correlation values are displayed within each cell of the correlation heat map, with color intensity indicating the strength of the relationships with variables (red is indicative of a strong positive correlation and blue a strong negative correlation).

To explore differences in response to priming, we constructed discrete networks for each family separately. When gene expression data were subset for family A (*n* = 18,868 genes), 15 modules were identified, seven of which were correlated with temperature (i.e., *p*‐value < 0.05; Figure 3). The two modules most correlated with temperature (i.e., module_FamA‐Royalblue_ and module_FamA‐Pink_) represent together 52% of genes of the whole dataset (9711 genes).

**FIGURE 4:**
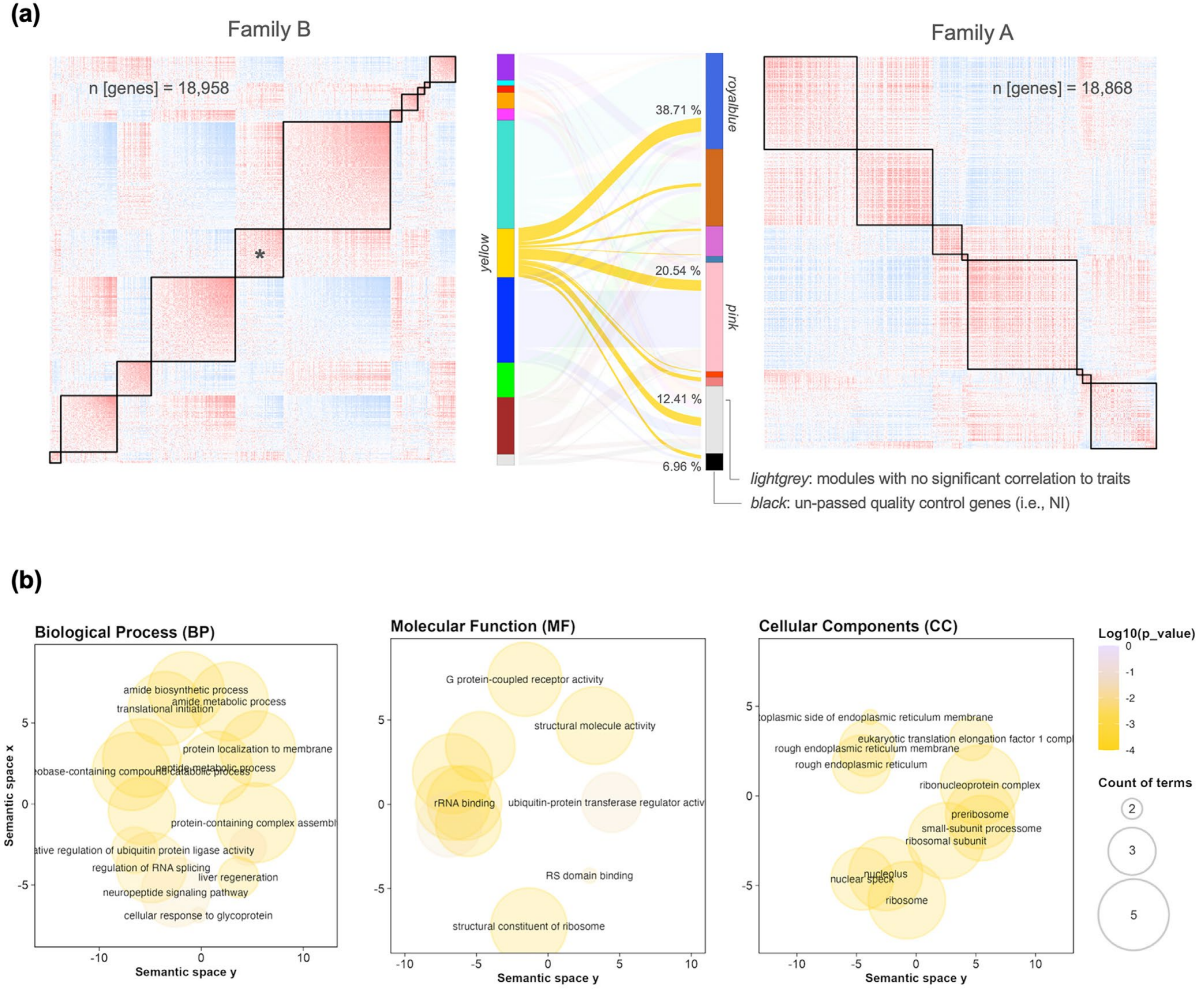
Module_FamB‐Yellow_. (a) Distribution of the genes in the identified co‐expression gene modules_FamA_. NI correspond to the genes that have not passed quality control to be included in the normalized input matrix (i.e., removed due to their too low variance, in the genotype C dataset). (b) Gene ontology scatterplot. Colors indicate the *p*‐value of enrichment according to the legend. The size of each bubble reflects the count of each term among the enriched term list.

When gene expression data were subset for family B (*n* = 18,958 genes),13 modules were obtained by clustering analysis, five of which were correlated with temperature, four with priming, and only one with both priming and temperature (i.e., module_FamB‐Yellow_; *ρ* = 0.69; *p*‐value < 0.01 Figure 3). Genes in this later module (2246 genes) show mostly enrichment for translation initiation, mRNA and peptide and metabolic process, regulation of mRNA splicing, protein‐containing complex assembly, and protein localization to membrane (Figure 4b). Cellular components (CC), molecular functions (MF), and GO annotations referred almost exclusively to endoplasmic reticulum, ribosome, translation elongation factor, rRNA and mRNA binding activity. All detailed GO annotations are available in Table S2.

### 3.7 Network Preservation

Of the 10 modules identified in family B, eight displayed strong evidence of preservation in family A (*p*‐values < 0.01). Two modules, module_FamB‐Cyan_ and module_FamB‐Yellow_, diverged in weight network statistic (*p*‐values = 0.11 and 1, respectively), while only the module_FamB‐Yellow_ (the only module significantly correlated with priming and temperature), showed significant divergence for coherence network statistic (*p*‐value = 1; Figure S6). Genes in this module mainly split in two modules in family A with opposite eigengene vectors, namely the module_FamA‐Royalblue_ (*n* = 841, ~39%) and module_FamA‐Pink_ (*n* = 455, ~21%; see Figure 4a), which were significantly positively and negatively correlated with temperature, respectively. Moreover, we can see that 169 genes (~7%) were absent from family A dataset because of low variance (variance < 0.05) from the normalized (vst) expression matrix (module_FamA‐Black_ Figure 4a).

### 3.8 Identification of Candidate Biological Processes Affected by Early‐Life Thermal Priming

**FIGURE 5:**
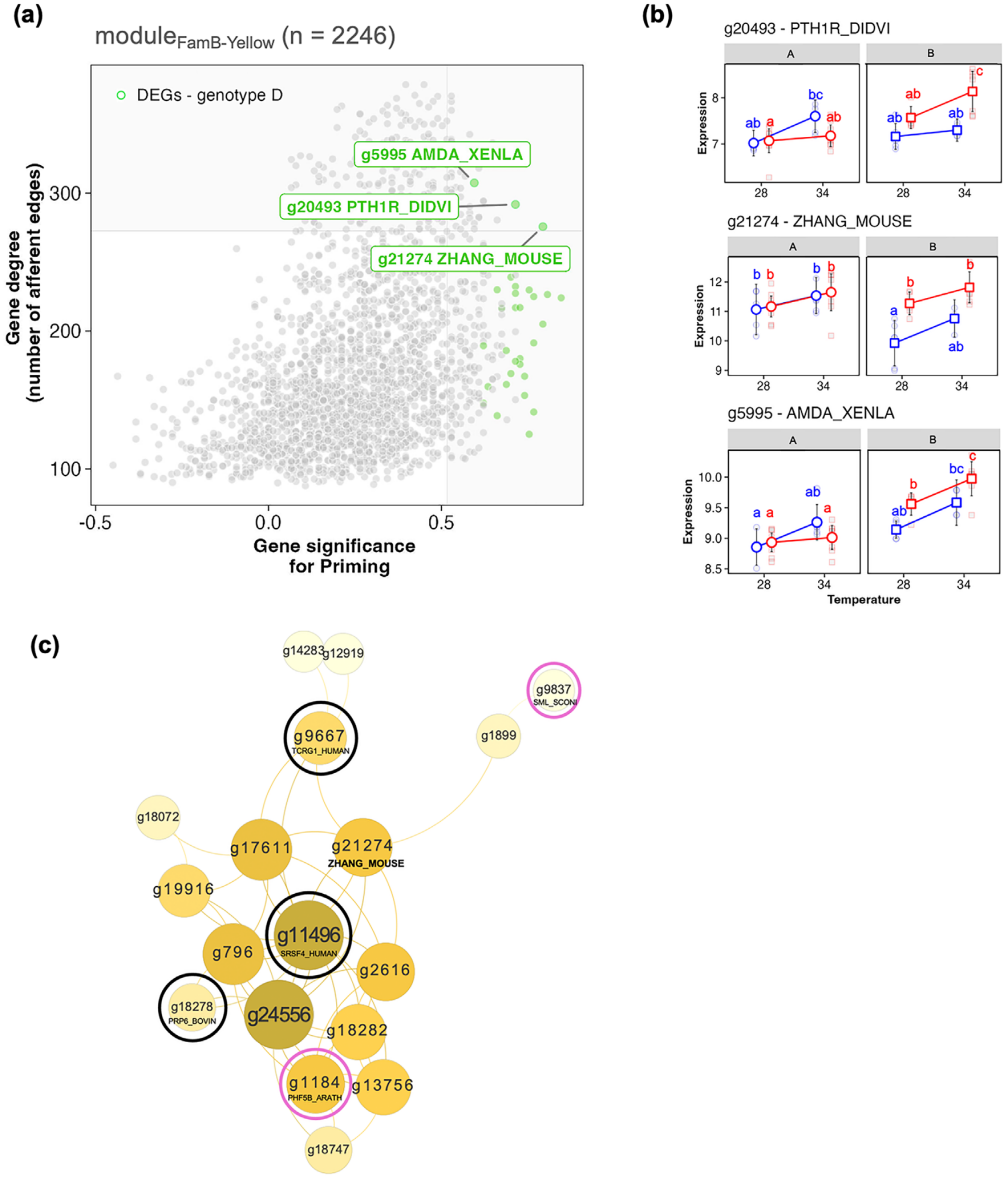
Candidate genes and evidence of gene network restructuration. (a) Scatterplot of gene degree (i.e., measure of centrality; number of afferent edges) vs. gene significance for priming—in the yellow module (family B). Gray area represents top 10 quantile of each metric. Greens dots represent DEGs previously identified with LRTs. (b) Panels display the mean ± 95 CI vst gene expression values of the three candidate genes, between families, early‐life thermal background, and later‐life temperature exposure, with letters for Tukey's a posteriori significant differences (*p*‐value < 0.05). (c) Network representation of the g21274 top 1% most correlated genes. Node size (gene) is represented proportional to its associated degree. Dark and pink circles inform for un‐passed quality control (i.e., NI), and pink module affiliation (i.e., negatively correlated with temperature) genes in the family A, respectively.

To generate a fine‐tuned and robust list of candidate genes—i.e., having a potentially significant role in the family × priming thermal tolerance variation—we first crossed (i) the list of DEGs, the (ii) top 10 most correlated genes with priming (WGCNA output), and the (iii) top 10 most ‘hub’ genes (from NetRep output) of the module_FamB‐Yellow_. Three genes were found which are functionally implicated in the biosynthesis of signaling peptides (g20493), CREB/ATF bZIP transcription factor, (g21274) and parathyroid hormone (PTH)/PTH‐related peptide receptor (g5995). Associated proteins are available in Table S2. Gene expression values revealed upregulation of these genes under heat stress (34°C), with significant variation across early‐life thermal background in family B (see in Figure 5b). Indeed, while g20493 expression was significantly higher in primed vs. naïve under heat stress (Tukey's HSD; *p*‐value < 0.05), g21274 expression was significantly higher in primed vs. naïve but under control condition (Tukey's HSD; *p*‐value < 0.05). And for g5995, expression of primed individuals was significantly higher under heat stress, than under control condition (Tukey's HSD; *p*‐value < 0.05). Network topography of the top 1 correlated node (*n* = 18) of g21274 revealed the presence of un‐passed control variance genes (Figure 5c black circles; ‘NI’) and genes included in module_FamA‐Pink_ (pink circles; down‐regulated with temperature). Among the 18 genes, the 3 NI genes were found to be implicated in pre‐mRNA splicing and transcription elongation regulator 1.

## 4 Discussion

Evidence for the long‐lasting effects of developmental thermal environments on later‐life phenotypes has been documented in several ectothermic taxa. Although this adaptive capacity holds promise for helping species cope with rapid global warming, the underlying molecular mechanisms are complex, varying across species, geographical regions, and thermal regimes (Kellermann and Sgrò 2018). As a result, the extent to which these effects contribute to meaningful long‐term adaptive responses remains unclear. In this study, we examined the capacity of two bi‐parental progenies (family A and B) of tropical bivalve, *Pinctada margaritifera* , to enhance spat's thermal tolerance (lethal stress) and performance (sublethal stress), through developmental thermal priming. Finally, the molecular pathways of the sublethal stress response were investigated using transcriptomic approaches, in an attempt to understand developmental plasticity mechanisms. The main molecular pathways of heat stress response were conserved across families and independent of the early‐life priming treatment. Nevertheless, the network preservation approach allowed further characterizing the subtle, nested early‐life environmental “memory” mediated through the network of gene expression reorganization.

### 4.1 Family‐Specific Effects of Developmental Temperature on Later‐Life Thermal Tolerance

This study shows that early‐life thermal priming affects later thermal tolerance in a genotype‐dependent manner. In family B, priming enhanced tolerance (LT50 = 9 days), while in family A, it reduced it (LT50 = 1 day). Under heat stress, primed individuals from family A showed increased respiration rates, suggesting higher thermal sensitivity, whereas rates in family B remained stable. This response may indicate either adaptive metabolic flexibility or impaired molecular buffering (Ritchie 2018). In the case of the adaptive metabolic flexibility, thermodynamic or passive adjustments in metabolism, would allow the organism to respond efficiently to changing conditions. By contrast, impaired molecular buffering would indicate incomplete compensatory mechanisms, limiting the organism's ability to maintain homeostasis under stress (Havird et al. 2020). Survival patterns support the former in family B and the latter in family A. Since mortality rate was somehow higher in primed family B, it could be argued that selective process might have favored a thermo‐tolerant phenotype in family B. However, putative selection was not supported by genetic data, with no large effect SNPs, polygenic signals nor nucleotide diversity analyses supporting priming‐induced changes in alleles frequencies. This result should nevertheless be taken with caution since we only examined genetic variants in coding regions. Furthermore, the low survivorship at metamorphosis typical of r‐strategists has been linked to a high genetic load and the accumulation of mildly deleterious alleles in oysters (Harrang et al. 2013; Plough et al. 2016), making the dynamics of selection particularly difficult to disentangle. Studies in mollusks have shown that allele frequencies can shift across developmental stages under control versus stress conditions (Bitter et al. 2019; Garland et al. 2022; Pespeni et al. 2013; Plough et al. 2016; Thomsen et al. 2017). While factors such as carry‐over effects, unequal family representation, and variable recombination rates might have been overlooked in those previous studies, allelic shift across developmental stages may contribute to obscuring the identification of polygenic signatures of deleterious effects and hinder our ability to accurately predict evolutionary trajectories (Ashlock et al. 2024). In ectotherms, persistent effects have received limited attention compared to rapid and transient response 23 studies out of 150 papers; reviewed in Pottier et al. (2022). Meta‐analyses reveal however large variation in effect size across species and studies, but also suggests that embryos raised either in stress or control temperature upon hatching tend to display reduced thermal tolerance (Pottier et al. 2022). Our results further nuance the overly simplistic view of beneficial “hormetic priming” and highlight the need to integrate genetic responses when assessing stress effects. In particular, the genetic background, often overlooked, may itself blur or modulate the observed signals (e.g., see Scott and Johnston 2012). This idea is in line with the conception of the genotype as an “environmental response repertoire rather than a fixed developmental program” proposed by Sonia (2017).

### 4.2 Conserved Effects of Heat Stress on Gene Expression

To investigate the underlying molecular mechanisms of such divergent phenotypes, we investigated genome‐wide gene‐expression profiling under sublethal heat stress following a 5 month common‐garden period. First, we showed that families accounted for a large proportion of gene expression variation. Second, our data revealed that while temperature response (control vs heat‐stress) was conserved between families (A vs. B), the effect of early‐life thermal environment (naïve vs. primed) differed between them. Specifically, differential expression analysis identified significantly more genes explained by the temperature effect (*n* ~ 5000) than by the priming and/or interaction (*n* ~ 100), for both families. The GO terms enrichment analysis of the modules correlated to temperature revealed the enrichment of highly conserved molecular pathways underlying cellular responses to thermal stress. These mechanisms, well documented in the literature (e.g., heat shock response (HSR); Gross 2004), are shared across diverse organisms, including mollusks. The observation that transcriptomic responses to temperature are modulated by thermal priming in a family‐dependent manner suggests that environmental “’memory” operates in discrete and complex ways. This underscores the importance of integrative‐omics approaches to unravel the subtle and nested effects of early‐life thermal conditions.

### 4.3 Network‐Based Approach to Explore Early Environmental Stress “Memory”

To generate biologically relevant gene co‐expression networks, recent frameworks have emphasized the importance of subdividing large datasets. This approach has been particularly valuable in molecular medicine for distinguishing between healthy and diseased states (e.g., Tommasini and Fogel 2023). Building on this, we generated family‐dependent gene co‐expression networks, which were subsequently tested for preservation. Our results revealed that a single gene module (in family B) was not preserved in family A (i.e., “Yellow”). Given that the expression of these nonpreserved genes was significantly positively correlated with both priming and temperature in family B, while being broadly distributed across co‐expression clusters in family A (responding differently to temperature effect), our results suggest that early‐life thermal priming in family B may have led to a (re)structuring of this gene expression network, likely conditioning the subsequent heat response.

Enrichment analysis of the GO terms for these non‐preserved genes (*n* = 2246) revealed a clear association with cellular components related to the rough endoplasmic reticulum. In contrast, the regulatory pathways were more diverse, including the assembly of protein complexes, protein localization to membranes, and the metabolic processes associated with mRNA. By combining network preservation with clustering and differential analyses, we obtained a relatively small set of genes (*n* = 3) encoding for biosynthesis of signaling peptides (g20493), CREB/ATF bZIP transcription factor (g21274), and parathyroid hormone (PTH)/PTH‐related peptide receptor (g5995).

The CREB/ATF family of transcription factors are key regulators in the Unfolded Protein Response (UPR; Walter and Ron 2011) signaling cascade pathway (Wang et al. 2019). Due to its alignment with the distinct patterns observed in cellular components (as revealed by GO term enrichment) and its well‐established role in thermal stress response, CREB/ATF bZIP transcription factor emerges as a particularly interesting candidate for further investigation. Nevertheless, it encompasses a broad spectrum of proteins involved in the complex UPR, participating on multiple cascading, feedback, and pleiotropic effects. Hence, a comprehensive understanding of this pathway dysregulation remains challenging in mollusks. By modeling top 1% most correlated genes within the network (in family B), we showed that another transcription factor, namely the *Transcription elongation regulator 1*, is totally decoupled between genotypes (i.e., no variation in family A). This transcription factor has been frequently identified in heat‐stress responses across kingdoms (e.g., from plants to mammals; Szádeczky‐Kardoss et al. 2022; Vera et al. 2014). Taken together, these results suggest that the enhanced heat response of primed individuals in family B is likely related with a network (re)structuration of gene‐regulation mechanisms implicated in the UPR. Indeed, by combining clustering, differential analyses, and thermal tolerance phenotypes, we uncovered the significant biological importance of a small subset of genes within complex cellular systems, thereby increasing the resolution of previous network‐based observations (Ripley et al. 2023). This work further strengthens the evidence for the wide range of processes underlying environmental “memory” (potentially synergetic, e.g., DNA chromatin methylation) and highlights that additional efforts are warranted to fully elucidate the drivers of developmental plasticity. Further work should determine if UPR modulation is a common feature, across multiple genotypes, of hormetic priming or if the mechanisms are also largely genotype‐dependent.

### 4.4 Implications for Aquaculture and Future Directions

Aquaculture is an economic sector particularly sensitive to global change; hence, maintaining stock integrity and performance under climate change projections is paramount. Efforts have been invested to develop programs for selecting highly‐tolerant genotypes or strains (Liu et al. 2022). However, the risks of losing standing genetic variation (D'Ambrosio et al. 2019), trade‐offs between traits (e.g., beneficial change in the heat stress survival may be linked to detrimental change in another trait, such as disease resistance; Gallardo‐Hidalgo et al. 2021; Pörtner et al. 2006), along with the risk of large genetic homogenization due to large inter‐island human‐derived transfers (Gueguen et al. 2016; Lemer and Planes 2012; Raapoto et al. 2023) pose a serious concern on long‐term sustainability. Here, we aimed at generating environmentally tailored phenotypes through hormetic priming, a strategy that, while minimizing the effects of domestication and genetic selection, could support restoration and breeding programs under global change. However, our findings suggests that much remains to be experimentally investigated before endorsing irreversible “hormetic priming” as the “silver bullet” for ecological conservation and aquaculture enhancement. Indeed, fundamental questions need to be addressed:

(i) *How and when applying early stimulation?* (ii) *Is beneficial hormesis more common than detrimental across genotypes?* (iii) *Does hormetic priming for temperature have trade‐off effects (*i.e., *the cost of plasticity)?* (iv) *Does thermal‐tolerance in acute lethal stress reflect tolerance under more environmentally realistic scenarios?*

Marine bivalves' early‐developmental stages undergo rapid and successive changes in endogenous physiological processes (e.g., metabolism, hormonal regulation, cell division and differentiation), along with highly variable DNA methylation patterns (Fellous et al. 2022), offering an ideal window for developmental imprinting (Burton and Metcalfe 2014; Fawcett and Frankenhuis 2015). Along with the results of this study, these elements are in line with the relevance of the critical window of embryogenesis for the “*when*”. As for the “*how*”, positive hormetic effects from low/intermediate doses of stress have been widely demonstrated across taxa (Costantini et al. 2014), while higher doses tend to produce injury/negative effects (e.g., see Glass et al. 2023). Supporting this, the *acclimation hypothesis* predicts beneficial developmental plasticity if organisms are able to anticipate ensuing environmental changes, by means of autocorrelated cues (Levins 1964; Lande 2009). Cues at the extreme, or exceeding the specie's range of tolerance are more likely conducive to environment mismatches, and thus maladaptive developmental responses (Diaz et al. 2021), as they have never been experienced in natural systems. An increasing body of evidence indicates that global warming has been accompanied not only by a rise in mean temperatures, but also by substantial changes in thermal variability since the pre‐industrial era (e.g., reported in Kefford et al. 2022). Importantly, these changes are not uniform across climate zones. Over a 40‐year period, the amplitude of diel temperature variability increased by approximately 1.4°C in polar regions, 1.0°C in temperate regions, and 0.3°C in tropical regions (Wang and Dillon 2014). In the context of Janzen's hypothesis, if thermal variability continues to increase in historically thermally stable environments such as the tropics and polar regions, extreme thermal cues during development would become more frequent, potentially undermining the adaptive value of developmental plasticity and increasing the risk of phenotype–environment mismatches. By contrast, temperate species, which have evolved under greater historical thermal variability, may be better able to capitalize on developmental plasticity under future climatic conditions.

### 4.5 Study Limitations and Perspectives

This study provides valuable insight into genotype‐specific responses to developmental thermal priming in *Pinctada margaritifera* . However, important limitations must be acknowledged. The use of only two bi‐parental crosses constrains the generalizability of our findings. The observed differences in mortality between families also limit our ability to fully disentangle the effects of priming from those of genotype alone. Moreover, unintentional selection may have occurred during early development, particularly given the higher mortality in primed individuals, notably in family B. Although genetic data did not support strong selection signals, our analysis was restricted to coding regions and may have missed changes in regulatory or noncoding elements.

Despite these limitations, several lines of evidence strengthen our conclusions. Naïve individuals from both families displayed nearly identical thermal performance, in contrast to the diverging responses observed under priming conditions. This suggests that spat energy allocation or overall physiological “quality” was initially comparable across families, reinforcing the idea that the observed differences are primarily driven by the interaction between genotype and early thermal environment. Furthermore, molecular and physiological analyses consistently aligned with these phenotypic differences, supporting the existence of a genotype‐dependent molecular ‘memory’ of early‐life thermal stress. These findings suggest that early‐stimulation endpoints are strongly influenced by genotype, with some genotypes benefiting from priming while others may be adversely affected. This reinforces the need for caution when applying hormetic priming strategies in aquaculture, as such approaches, if not carefully managed, could inadvertently favor certain genotypes over others and reduce standing genetic variation, mirroring the risks of traditional selective breeding programs. To minimize the influence of direct nongenetic parental effects, broodstock from the same origin populations and sampled in the field at the same date were maintained for 7 years under similar conditions where they never experienced critical upper thermal limit and had the opportunity to engage in numerous gametogenesis and spawning events. Nevertheless, the long‐term maintenance under common‐garden conditions is unlikely to have fully attenuated the divergent environmental histories experienced by breeders prior to collection. Such pre‐exposure effects may therefore have persisted and subsequently manifested as trans‐ and intergenerational epigenetic inheritance. We also cannot exclude additional sources of variation such as parental age effects, which may also contribute to the observed phenotypic outcomes (Boulais et al. 2015; Donelson et al. 2018; Dupoué et al. 2024). Together, these results underscore the complexity of developmental plasticity and the need for further experimental work, involving larger numbers of families and a broader range of environmental conditions, to robustly assess the potential and limitations of hormetic priming in conservation and aquaculture.

## 5 Conclusions

In this study, the effect of embryonic thermal priming on later‐life phenotypes was explored using in depth‐transcriptomic analyses. Our results reveal family‐specific effects of priming, significantly enhancing thermal tolerance in one family while deteriorating it in the other. Variation across the transcriptome was mostly driven by highly conserved heat response genes. The network preservation approach provided an opportunity to characterize the subtle, nested environmental ‘memory’ mediated through network reorganization of gene expression, particularly in gene regulatory pathways involved in the Unfolding Protein Response (UPR). These results suggest for a reconsideration of the *beneficial acclimation hypothesis* and the potential for developmental plasticity to mitigate the impacts of ongoing ocean warming through improved husbandry practices.

To decipher the mechanisms underlying developmental plasticity, this experiment was designed to minimize as much as possible—within the constraints of zootechnical feasibility—the potential drivers of phenotypic variation. This was achieved through the use of controlled pedigree and recent life history of bi‐parental breeders (approximately 7 years of common‐garden maintenance prior to reproduction.), along with common‐garden laboratory conditions for the spat. While naive cohorts of both families exhibited similar responses to lethal thermal stress, our results do not exclude the potential involvement of additional indirect, non‐genetic factors in the ultimate outcomes of early‐life priming. We thus strongly encourage further research to explore a broader range of milder stresses and genotype combinations in order to provide solid results and provide valuable insights for aquaculture enhancement and guide resource management and conservation efforts.

## Author Contributions

**K. Lugue:** conceptualization (equal), formal analysis (equal), investigation (equal), writing – original draft (equal). **C. J. Monaco:** conceptualization (equal), methodology (equal), writing – review and editing (equal). **L. Benestan:** formal analysis (equal), writing – original draft (equal), writing – review and editing (equal). **E. Vigouroux:** resources (supporting). **M. Sham Koua:** resources (supporting). **J. Vidal‐Dupiol:** conceptualization (equal), supervision (supporting), writing – review and editing (equal). **G. Mitta:** methodology (supporting), supervision (supporting), writing – review and editing (supporting). **J. Le Luyer:** conceptualization (lead), formal analysis (lead), funding acquisition (lead), project administration (lead), supervision (lead), writing – original draft (equal), writing – review and editing (equal).

## Funding

This study received financial support from IFREMER through the PinctAdapt project.

## Disclosure

Benefits Generated: Benefits from this research accrue from the sharing of our data and results on public databases as described above.

## Conflicts of Interest

The authors declare no conflicts of interest.

## Supporting information


**Data S1:** ece373139‐sup‐0001‐DataS1.docx.

**Table S1:** ece373139‐sup‐0002‐TableS1.xlsx.

**Table S2:** ece373139‐sup‐0003‐TableS2.xlsx.

## Data Availability

Supporting Information figures and tables supporting this article are available in Data S1. Differentially expressed genes annotations and module_FamB‐Yellow_ GO terms enrichment can be found in the Tables S1 and S2, respectively. Raw sequence reads have been deposited in the European Nucleotide Archive (project accession: PRJEB85440; https://www.ebi.ac.uk/ena/browser/view/PRJEB85440). The code is publicly available on Zenodo and has been assigned a DOI: https://doi.org/10.5281/zenodo.18385792.
