# Supplementary material for: Embryonic Hormetic Priming Modulates Later‐Life Thermal Tolerance
Source: Ecol Evol. 2026 Mar 14;16(3):e73139. doi: 10.1002/ece3.73139 (PMC13093313; doi:10.1002/ece3.73139)
Supplement: Supplementary file 1 — Data S1: ece373139‐sup‐0001‐DataS1.docx. [file ECE3-16-e73139-s003.docx]

## **1 | Supplementary materials and methods**

### **1.1 | Experimental tanks**

The experimental set up consisted of independent, 29.2-L open flow tanks, supplied with 10-µm filtered and UV-treated seawater (flow rate = 20 L h^-1^, turnover rate = 68% h^-1^). Food (1:1 mix of *Tisochrysis galbana* and *Chaetoceros gracillis*) was continuously provided at 800 μm^3^ mL^−1^ (measured at the outlet of the tank), and controlled daily using an electronic particle counter (Multisizer3, Beckman Coulter; 100-μm aperture tube). Air stones allowed a well-oxygenated environment, and homogeneous concentration of phytoplankton cells. Overall, conditions recreated ambient seawater parameters (temperature 27°C, salinity 36 psu, pH_NBS_ 8.3, DO_2_ saturation 95%, photoperiod 14:10 D:L), which were daily checked (multi-Parameter WTW®). For thermal stress assays, seawater temperature was regulated using heaters (IKS^®^ Aquastar system) or chiller (TECO^®^), for warm (>28°C) or cold (<28°C) conditions, respectively.

### **1.2 | Metabolic and ingestion rate measurements and calculations**

Spats were randomly sampled from experimental tanks, and placed in individual closed-system respirometry chambers (120-mL volume), containing 1-µm filtered and oxygen-saturated seawater, and maintained by water bath at the corresponding treatment temperature in experimental tanks dedicated to this purpose. Once bivalves appeared to have started filtering (as indicated by open valves and the mantle edge and tentacles in an outstretched position, ~ 10 minutes), respiration measurements started, for a total duration of 1.5 hours. Measurements of oxygen concentration were done using an optical sensor coupled with an Opto-F1 UniAmp (Unisense®). Respiration rates (RR; mg O_2_ h^-1^) were corrected to a standard 1-g animal, using the formula:

*Ys = (Ws/We)^b^ × Ye (eq. S1)*

Where Ys is the corrected metabolic rate, Ws is the standard dry weight (1 g), We is the measured dry weight, Ye is the measured physiological activity, and b is the allometric coefficient. Based on Savina & Pouvreau (2004), we used b allometric coefficient of 0.75.

- 1. **| Genome-wide transcriptome plasticity**

In order to explore shifts in expression profiles, all 20,279 highly expressed genes were first transformed using a Principal Components Analysis (PCA) for genotype C and D, separately. A discriminant function was then built by defining early-life conditions as groups (naive = 1, primed = 2) in the control thermal treatment (28°C); using the adegenet v2.1.10 R package (Jombart et al., 2010) *.* The number of PCs used to create the function were chosen according to Kenkel & Matz, 2016. The function was then applied to the individuals under the heat stress (34°C), and plotted on the same axis as the control treatment response. Shifts magnitude induced by the heat stress were then inferred to each *Genotype* x *Priming* combination using the Markov chain Monte Carlo (MCMC) linear mixed model (MCMCglmm v2.35 R package; Hadfield, 2010). The final model was:

*DAPC individual scores (LD1)* *~ Priming + Priming: Treatment*

The *p* value was estimated as the absolute difference in magnitudes of *naïve:34* and *primed:34* using the sampled sets of parameters (i.e., length of arrows; Kenkel & Matz, 2016).

## **| Supplementary tables**

**Table S1**. Larval rearing data.

| **Date** | **ID familly** | **Nb eggs** | **Fertiliz- ation rate** |  | **3 – 24 hpf Incubation** | | |  | **Nb D-shape larvae (24 hpf)** | **Hatching rate** | **Survival rate** |
| --- | --- | --- | --- | --- | --- | --- | --- | --- | --- | --- | --- |
|  |  |  |  |  | **Initial eggs (nb)** | **Density (larve/mL)** | **Temperature (°C)** |  |  |  |  |
|  |  |  |  |  |  |  |  |  |  |  |  |
| 17/08/2022 | A | 1 924 000 | 86% |  | 250 000 | 3,57 | 31,6 |  | 110833 | 44% | 2% |
|  |  |  |  |  | 250 000 | 3,57 | 31,6 |  | 110000 | 44% | 5% |
|  |  |  |  |  | 250 000 | 3,57 | 27,8 |  | 111667 | 45% | 10% |
|  |  |  |  |  | 250 000 | 3,57 | 27,8 |  | 161667 | 65% | 17% |
|  |  |  |  |  |  |  |  |  |  |  |  |
| 21/09/2022 | B | 1 200 000 | 97% |  | 1 200 000 | 17,14 | 32 |  | 90000 | 8% | 11% |
|  |  |  |  |  | 1 200 000 | 17,14 | 28 |  | 390000 | 33% | 27% |
|  |  |  |  |  |  |  |  |  |  |  |  |

**Table S2.** List of genes of interest.

| **name** | **ID** | **protein** |
| --- | --- | --- |
| g1 | g20493 | PTH1R_DIDVI |
| g2 | g21274 | ZHANG_MOUSE |
| g3 | g5995 | AMDA_XENLA |

##
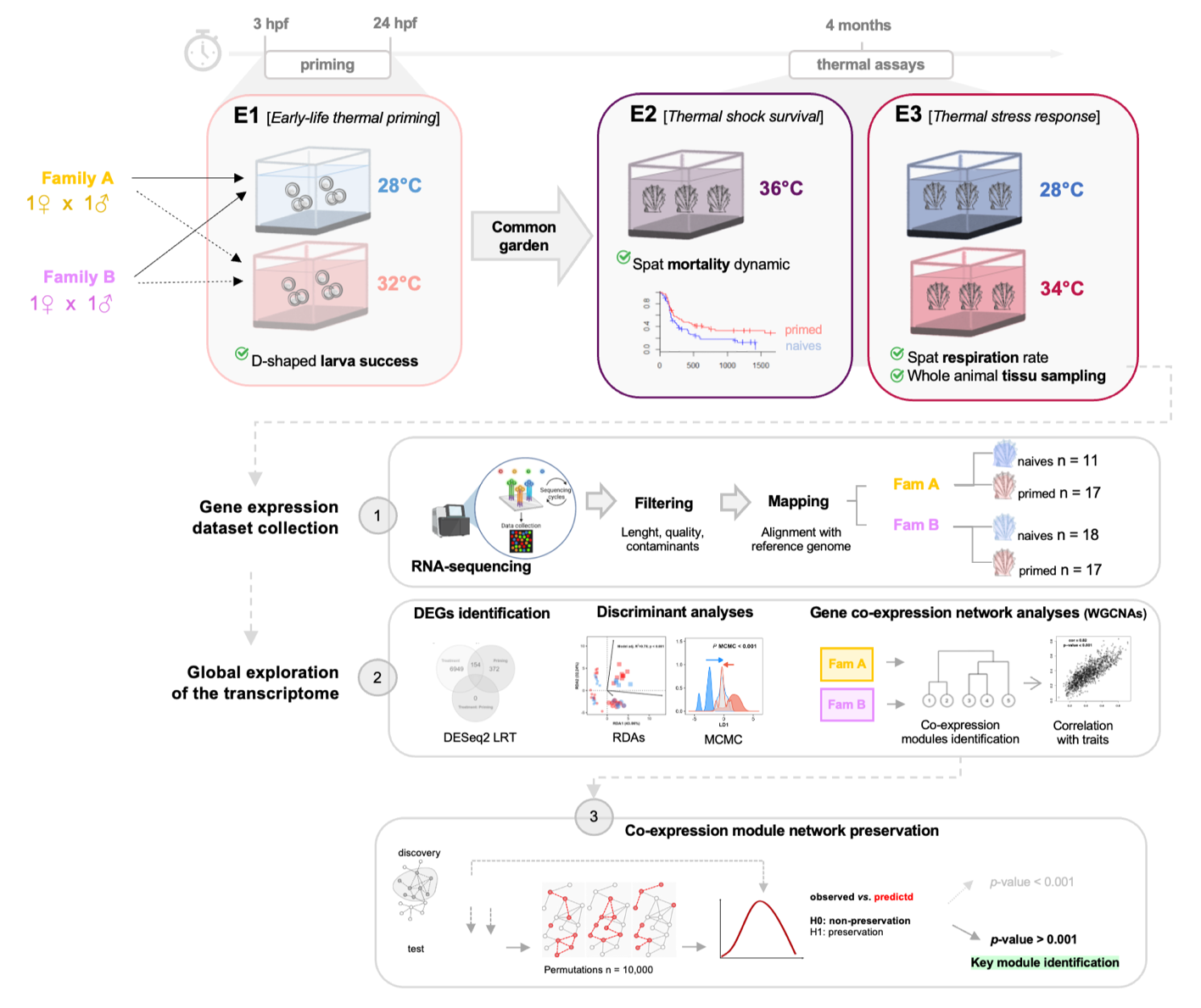
**3 | Supplementary figures**

**Figure S1**. Graphical abstract


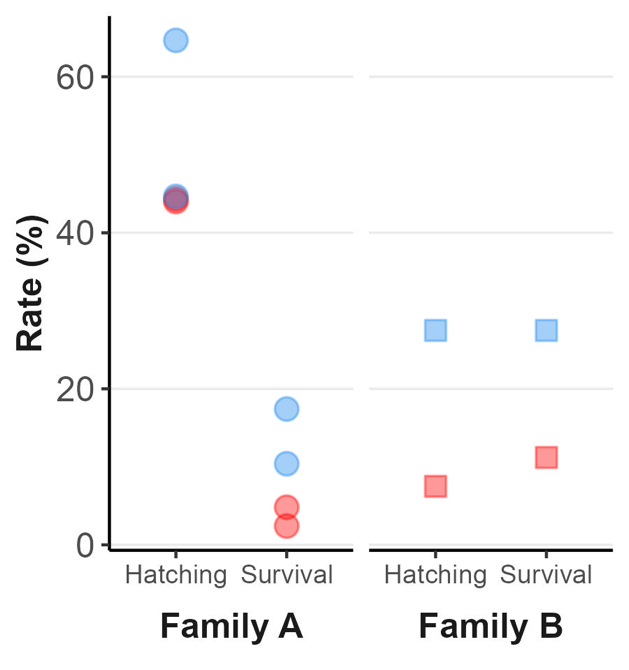
**Figure S2.** Percentage of hatching (number of D-shape larvae relative to the number of initial embryos) and larval rearing final survival (number of settled eye-spot larvae relative to the number of D-shape larvae), in a function of family and early-life thermal condition (blue: control 28°C; red: warm 32°C).


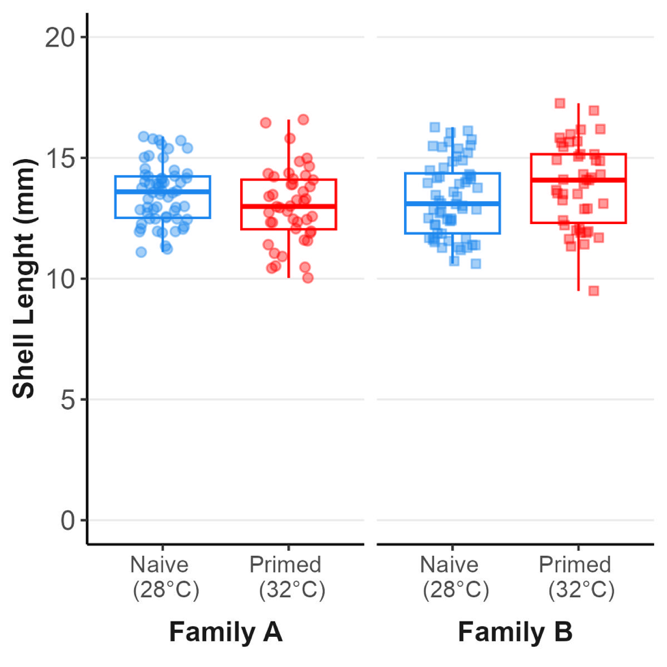


Figure S3. Shell length of individuals used for the E2 experiment.


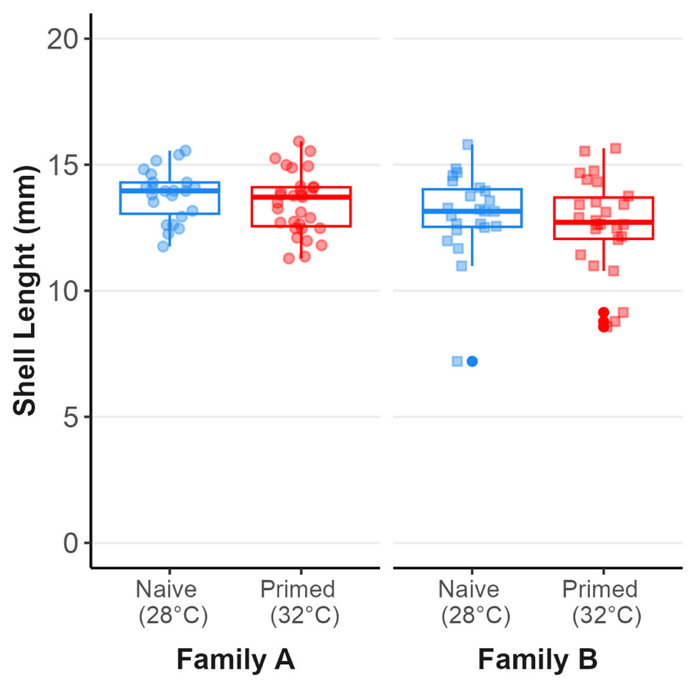


Figure S4. Shell length of individuals used for the E2 experiment.

**
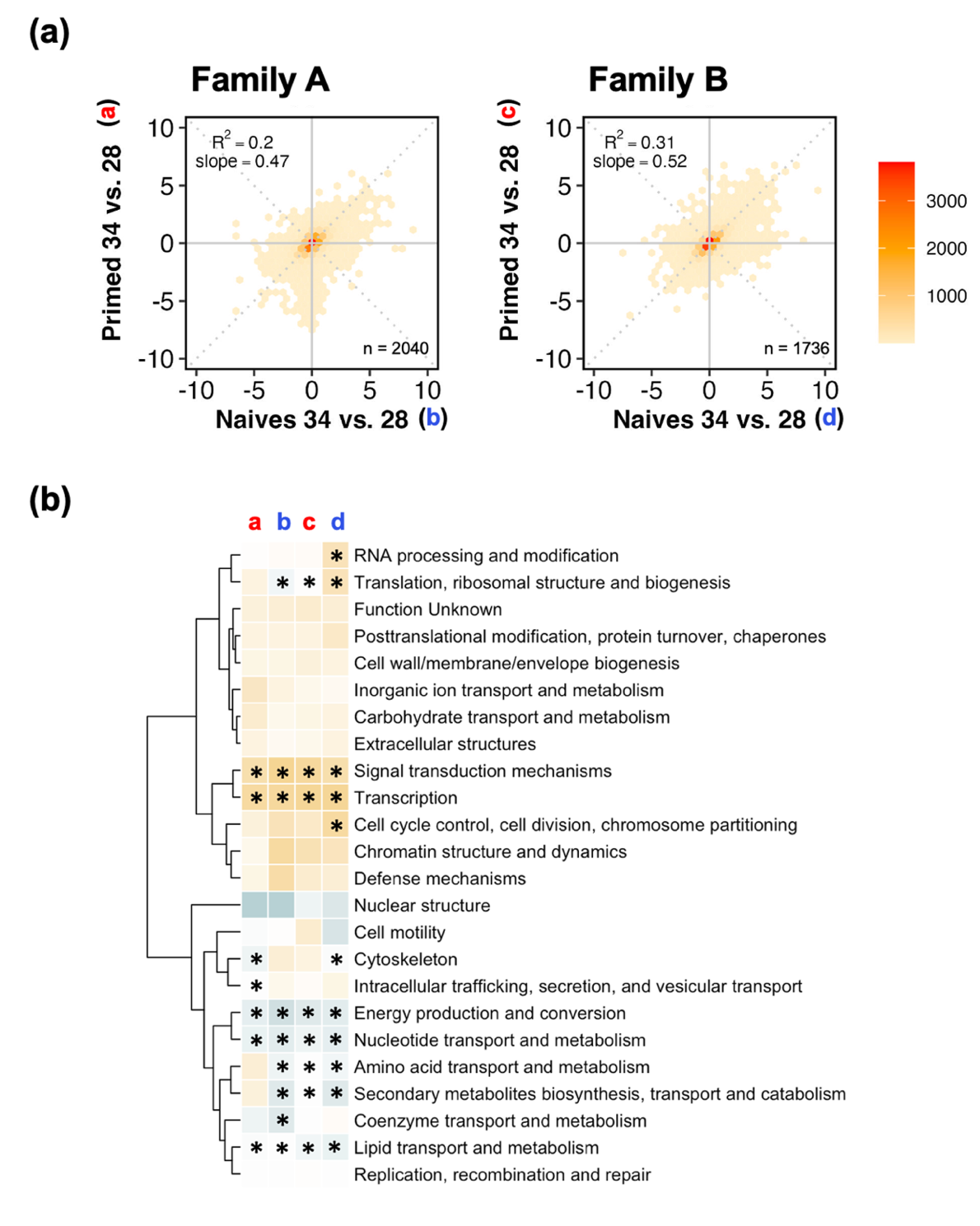
Figure S5.** Spats gene expression heat stress responses compared among datasets. (a) Per-gene log2-fold changes under control versus stressed treatment in primed individuals (y-axis) plotted against log2-fold changes under control versus stressed treatment in naive individuals (x-axis); for each genotype separately. The color of the plot represents the density of the points; with the highest density in red, and the lowest density. (b) Heat map of enrichment of KOG classes (rows) by differentially expressed genes (contrast models) in different datasets (columns).


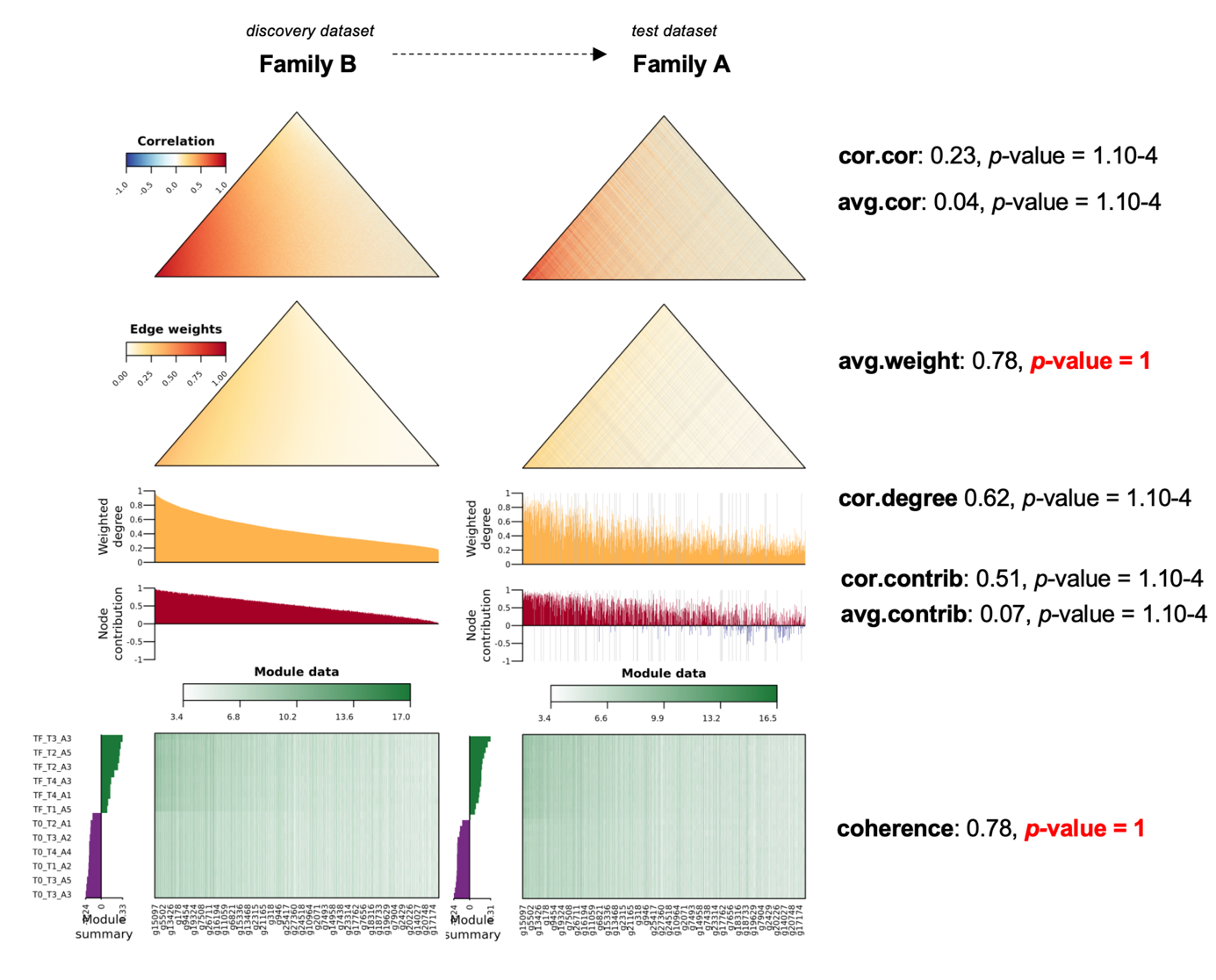


**Figure S6. Network topology of the yellow module (FamB-A).** On the left, network topology in the family B (discovery dataset). From top to bottom: heatmap of the correlation structure (Pearson correlation), heatmap of the interaction network edge weights, normalized weighted degree (calculated within the module and normalized by the maximum value), and node contribution (Pearson correlation between each probe and the summary expression profile). Probes are ordered by descending order of weighted degree. On the right, network topology of the yellow module in the family A (test dataset). Probes are ordered as in the family B. Grey bars denote probes either missing, or not passing quality control, in the family A (‘NI’).

## **4 | References**

Hadfield, Jarrod D. 2010. « MCMC Methods for Multi-Response Generalized Linear Mixed Models: The MCMCglmm R Package ». *Journal of Statistical Software* 33:1‑22. doi: 10.18637/jss.v033.i02.

Jombart, Thibaut, Sébastien Devillard, et François Balloux. 2010. « Discriminant Analysis of Principal Components: A New Method for the Analysis of Genetically Structured Populations ». *BMC Genetics* 11(1):94. doi: 10.1186/1471-2156-11-94.

Kenkel, Carly D., et Mikhail V. Matz. 2016. « Gene Expression Plasticity as a Mechanism of Coral Adaptation to a Variable Environment ». *Nature Ecology & Evolution* 1(1):1‑6. doi: 10.1038/s41559-016-0014.

Ritchie, Scott C., Stephen Watts, Liam G. Fearnley, Kathryn E. Holt, Gad Abraham, et Michael Inouye. 2016. « A Scalable Permutation Approach Reveals Replication and Preservation Patterns of Network Modules in Large Datasets ». *Cell Systems* 3(1):71‑82. doi: 10.1016/j.cels.2016.06.012.

Savina, Marie, et Stéphane Pouvreau. 2004. « A Comparative Ecophysiological Study of Two Infaunal Filter-Feeding Bivalves: Paphia Rhomboı̈des and Glycymeris Glycymeris ». *Aquaculture* 239(1‑4):289‑306. doi: 10.1016/j.aquaculture.2004.05.029.
